# RAD-Seq-Based High-Density Linkage Maps Construction and Quantitative Trait Loci Mapping of Flowering Time Trait in Alfalfa (*Medicago sativa* L.)

**DOI:** 10.3389/fpls.2022.899681

**Published:** 2022-05-26

**Authors:** Xueqian Jiang, Tianhui Yang, Fan Zhang, Xijiang Yang, Changfu Yang, Fei He, Ruicai Long, Ting Gao, Yiwei Jiang, Qingchuan Yang, Zhen Wang, Junmei Kang

**Affiliations:** ^1^Institute of Animal Science, Chinese Academy of Agricultural Sciences, Beijing, China; ^2^Institute of Animal Science, Ningxia Academy of Agricultural and Forestry Sciences, Yinchuan, China; ^3^Department of Agronomy, Purdue University, West Lafayette, IN, United States

**Keywords:** alfalfa, flowering time, genetic map, QTL, RAD-seq, RNA-seq

## Abstract

Alfalfa (*Medicago sativa* L.) is a perennial forage crop known as the “Queen of Forages.” To dissect the genetic mechanism of flowering time (FT) in alfalfa, high−density linkage maps were constructed for both parents of an F1 mapping population derived from a cross between Cangzhou (P1) and ZhongmuNO.1 (P2), consisting of 150 progenies. The FT showed a transgressive segregation pattern in the mapping population. A total of 13,773 single-nucleotide polymorphism markers was obtained by using restriction-site associated DNA sequencing and distributed on 64 linkage groups, with a total length of 3,780.49 and 4,113.45 cM and an average marker interval of 0.58 and 0.59 cM for P1 and P2 parent, respectively. Quantitative trait loci (QTL) analyses were performed using the least square means of each year as well as the best linear unbiased prediction values across 4 years. Sixteen QTLs for FT were detected for P1 and 22 QTLs for P2, accounting for 1.40–16.04% of FT variation. RNA-Seq analysis at three flowering stages identified 5,039, 7,058, and 7,996 genes that were differentially expressed between two parents, respectively. Based on QTL mapping, DEGs analysis, and functional annotation, seven candidate genes associated with flowering time were finally detected. This study discovered QTLs and candidate genes for alfalfa FT, making it a useful resource for breeding studies on this essential crop.

## Introduction

Alfalfa (*Medicago sativa* L.) is an important forage species with high yields, quality, and adaptability. This species has a wide distribution all over the world (Radovic et al., 2009). China is one of the largest producers of alfalfa with a long history of its cultivation and utilization for livestock. However, the increasing demands for livestock production pose a challenge for the alfalfa industry (Bai et al., 2018), mainly due to a lack of alfalfa cultivars with a wide range of adaptation. This obstacle has been severely limiting the expansion of alfalfa cultivation and production in China. Until 2017, approximately 71% of the alfalfa was grown in the four provinces of northwestern China (Xinjiang, Inner Mongolia, Gansu, and Shaanxi). Therefore, it is crucial to develop alfalfa cultivars with improved yield and adaptability to meet the growing needs of alfalfa production.

Flowering time (FT), a major fitness component, is a key trait for the seasonal and geographical adaptation of flowering plants (Kooyers, 2015). FT is a signal that allows plants to change from vegetative growth to reproductive growth (Srikanth and Schmid, 2011), which plays an important role in alfalfa biomass yield. Premature flowering often leads to a shorter vegetative growth stage that results in reduced biomass yield, whereas delaying the flowering could increase plant yield and biomass of perennial crops, including alfalfa. Given the importance of FT, it has been suggested that the development of the novel varieties with modified FT may result in higher biomass production due to a better adaptation of plants to local environments and climate conditions (Schiffers et al., 2013).

Flowering time is a complex quantitative trait determined by genotype, environment, and the interactions of genotype and environment (Koornneef et al., 1998; Upadhyaya et al., 2015). Previous research showed that shifting FT-related genes enables plants to gain a wider adaptability (Diaz et al., 2012; Weller et al., 2019; Lu et al., 2020). For instance, *Tof11* and *Tof12* delayed FT under long photoperiods and improved plant adaptation to higher latitudes during soybean domestication (Lu et al., 2020). In addition, the mechanism of flowering time has been well studied in model plants like Arabidopsis and in crop species such as rice (*Oryza sativa* L.) (Hori et al., 2016; Kinoshita and Richter, 2020). Numerous candidate genes have been identified and their intricate regulatory networks are being deciphered, including *FLOWERING LOCUS T* T (*FT*), *CONSTANS* (*CO*), and FLOWERING LOCUS C (*FLC*)/mads affecting FLOWERING (*MAF*) (Song et al., 2013; Kinoshita and Richter, 2020). *FT*-like genes are involved in photoperiod sensing, and homologues of *FT* have also been identified to control flowering time in several legume species. For example, five homologues of *FT* were characterized in *Medicago truncatula*, two of which were flowering inductors (Hecht et al., 2011; Laurie et al., 2011). Five homologues were detected in alfalfa, but only *MsFTa1* was found to be a flowering inducer (Lorenzo et al., 2020). Nevertheless, no functional homologues of *CO* were identified in *Medicago truncatula* (Wong et al., 2014), suggesting that the *CO*-like genes were not conserved in legumes, including alfalfa.

Recently, RNA-sequencing (RNA-seq) has been widely applied in various species due to the rapid advancement of the technology and decreased price. However, through RNA-seq, a large quantity of differentially expressed genes (DEGs) are usually identified. Therefore, the strategy of QTL mapping combined with RNA-seq is considered to be a promising method to rapidly identify potential candidate genes. For example, integration of RNA-seq and QTLs mapping have been carried out to identify candidate genes associated with cadmium tolerance in barley (Hordeum vulgare) (Derakhshani et al., 2020), genes for pod number variation in rapeseed (*Brassica napus* L.) (Ye et al., 2017), and high-temperature stress-responsive genes in tomato (*Solanum lycopersicum* L.) (Wen et al., 2019).

As alfalfa is a long-day plant, its flowering is affected by both photoperiod and temperature (Major et al., 1991; Lorenzo et al., 2020). Although some FT-related QTLs have been investigated in alfalfa (Adhikari et al., 2019; Zhang et al., 2020), the genetic basis of flowering time remains largely unexplored. In previous studies, due to the lack of an alfalfa reference genome, it was difficult to construct high−density linkage maps and compare QTLs in different mapping populations. All of these studies were made feasible by the availability of a high-quality, chromosome-level alfalfa genome (Chen H. et al., 2020). RAD-seq is an economical and useful method of generating SNP markers across the whole genome (Huang et al., 2017; Lee et al., 2019). Therefore, the objectives of this study were to construct linkage maps using genome-wide SNP markers based on RAD-seq, detect the QTLs related to FT, and identify candidate genes by integrating the QTLs and RNA-seq.

## Materials and Methods

### Plant Materials and Phenotyping

An F_1_ population consisting of 150 progenies was developed by crossing a tetraploid early flowering and low forage yield alfalfa landrace (Cangzhou, CF000735, paternal parent, P1) with a late flowering and high forage yield cultivar (Zhongmu NO.1, CF0032020, maternal parent, P2). The seeds of the F_1_ population were planted and the clones of progenies and two parents were initially propagated in a greenhouse in 2012 in the Chinese Academy of Agricultural Sciences Research Station in Langfang, Hebei Province (39.59°N, 116.59°E). In 2013, the cloned plants were transferred to the field in Langfang, Hebei Province (Zhang et al., 2019), and propagated again in 2016 to ensure genetic uniformity of all plant materials. The field experiment was a randomized complete block design with three replications, where one clone for each progeny and parents was planted in per replication.

In this study, FT was defined as the date of the first flower appeared in individual plants. It was recorded daily from the date when the first flower appeared in a plant until all plants flowered. We collected FT data across 4 years from 2017 to 2020. FT dates were expressed as photothermal units (PTU) using the method described by Grabowski et al. (2017). The PTU was estimated using the following formula;


$$dPTU={\left(\left(minT+maxT\right)/2--10\right)}^{*}dayL/24$$

where dPTU = daily PTU; minT = the minimum recorded temperature; maxT = the maximum recorded temperature; dayL = the number of hours between sunrise and sunset. PTU starts accumulating after 5 consecutive days with (minT + maxT) > 10°C. The information of minT and maxT was obtained from the climatological station,^1^ and dayL data was collected from the website.^2^

We estimated the PTU values using the least-squares mean (LS mean), performing for each year separately with PROC GLM (v. 9.4; SAS Institute, Cary, NC, United States). The best linear unbiased prediction (BLUP) values were calculated using the “lme4” package in R (Bates et al., 2014). The R package lme4 was also used to estimate the broad-sense heritability (H^2^) according to the following formula:


$$H^{2}\frac{\sigma_{g}^{2}}{\sigma_{g}^{2}+\left(\sigma_{r}^{2}+\sigma_{\varepsilon }^{2}\right)/r}$$

where *H*^2^ = broad-sense heritability; σ^2^_*g*_ = genotypic variance; σ^2^_*r*_ = block variance; σ^2^_ε_ = residual error variance; *r*, the numbers of blocks. The LS means of each year along with BLUP values were used as the phenotype data for QTL analysis.

### DNA Extraction and Sequencing

The information on DNA isolation and RAD library construction has been described in detail in our previous study (Zhang et al., 2019). Briefly, young leaf tissues from the parents and F_1_ progenies were collected and frozen in liquid nitrogen. DNA was extracted using the CWBIO Plant Genomic DNA Kit (CoWin Biosciences, Beijing, China) according to the manufacturer’s protocol. DNA concentrations were measured using a NanoDrop 2000 spectrophotometer (Thermo Fisher Scientific, Waltham, MA, United States). RAD library preparation and sequencing were performed at ORI-GENE (Beijing, China), using a Hi-seq X Ten (Illumina). The RAD sequences were deposited in the NCBI Sequence Read Archive (PRJNA503672).

### Reference-Based Single-Nucleotide Polymorphism Calling and Genetic Linkage Maps Construction

Sequencing data was filtered using trimmomatic software with default parameters (Bolger et al., 2014). Paired-end sequencing reads were mapped to the alfalfa reference genome (Chen H. et al., 2020) with BWA-MEM (Li, 2013). Samtools was used to translate SAM files to BAM files and then to sort BAM files using default parameters (Li et al., 2009). GATK4.0 was used to mark duplicate reads and detect single-nucleotide polymorphism (SNP) (McKenna et al., 2010). Furthermore, SNP data was filtered using Vcftools (Danecek et al., 2011) with the missing rate less than 50%, minor allele frequency greater than 0.05 and mean read depth greater than 5. Single-dose allele (SDA) markers with a ratio of less than 2:1 and missing data rates of ≤ 20% were retained for constructing genetic linkage maps (Li et al., 2014). The regression method of JoinMap 4.0^3^ was used to order the markers within the chromosomes (Stam, 1993). Kosambi map function was used to translate recombination frequency into map distances.

### Quantitative Trait Loci Analysis for Flowering Time

Combining the phenotypic data and the genetic linkage maps, QTLs for FT were identified in the F_1_ population using the Inclusive Composite Interval Mapping with an additive effect (ICIM-ADD) in the QTL IciMapping software^4^ (Meng et al., 2015). The mapping parameter of each step for ICIM-ADD was set at 1.0 cM, and the LOD threshold was set at 3.0. When the genetic locations of the QTLs (at a 99% significance level) overlapped in different years, they were defined as a single QTL. The QTLs detected for each parental map were drawn on linkage maps using MapChart 2.3 (Voorrips, 2002). QTLs were named as: *qFT* + linkage group no., or named as *qFT* + linkage group no. + an ordered number designating one of multiple QTLs in a single linkage group. For example, *qFT4.1-2* indicates the second QTL in the Chr4.1 linkage group.

### RNA-Sequencing Analysis

The flower samples were collected at three flowering stages (budding stage, BS; initial flowering stage, IFS and full flowering stage, FFS) from two parental alfalfa plants and immediately frozen in liquid nitrogen and stored at −80°C. Altogether, 12 flower samples were collected for all two replicates. Total RNA was isolated using the TRIZOL reagent (Invitrogen, CA, United States) according to the manufacturer’s protocol. High-quality total RNA samples were sent to the Novogene (Beijing, China) and sequenced with an Illumina HiSeq 2000 platform. The obtained RNA-Seq data were submitted to the Sequence Read Archive of the National Center for Biotechnology Information (NCBI) with the accession number PRJNA817856.^5^

Estimation of RNA-Seq data quality was performed by FastQC v0.11.9,^6^ and low-quality sequences were filtered by fastp v0.12.4 (Chen et al., 2018). All filtered clean reads were mapped to the XJDY reference genome using hisat2 v4.8.2 (Kim et al., 2019), and the alignments were sorted using SAMTools (Li et al., 2009). Read counts for each sample were generated using featureCounts v2.0.1 (Liao et al., 2014). DEGs with an adjusted *p*-value ≤ 0.0001 and expression level of |log2 fold change| ≥ 3 was performed with the R package DESeq2 (Love et al., 2014).

### Comparison With Previously Reported Quantitative Trait Loci and Potential Candidate Gene Identification

The sequences of flanking markers for each previously reported QTL were used for local blast (*E*-value of 1e^–5^) against the alfalfa reference genome (cultivar XinJiangDaYe) (Chen H. et al., 2020), which was downloaded online.^7^ TBtools software (Chen C. et al., 2020) was used to complete the following analysis. First, we identified the physical positions based on the flanking marker sequences of the QTLs with the function of –BLAST–BLAST GUI Wrapper–Several Sequences to a Big File. Then we extracted genes on the alfalfa genome based on the overlapped physical positions in different genetic backgrounds (function: –Sequence Toolkit–GFF3/GTF Manipulate–GXF Regoin Overlap). DEGs within QTL intervals were annotated based on BLSAT search in NCBI,^8^ Ensembl,^9^ and eggNOG-mapper.^10^

### Quantitative Real-Time PCR

The RNA-seq data were validated using quantitative real-time PCR (qRT-PCR). On a 7500 Real-Time PCR System (Applied Biosystems, CA, United States), qRT-PCR was implemented in triplicate for each sample of two parents at three flowering stages using the SYBR Premix Ex Taq (Takara, Japan). The relative gene expression level was calculated by the 2^–ΔΔCt^ method.

## Results

### Phenotypic Data Analysis

**FIGURE 1:**
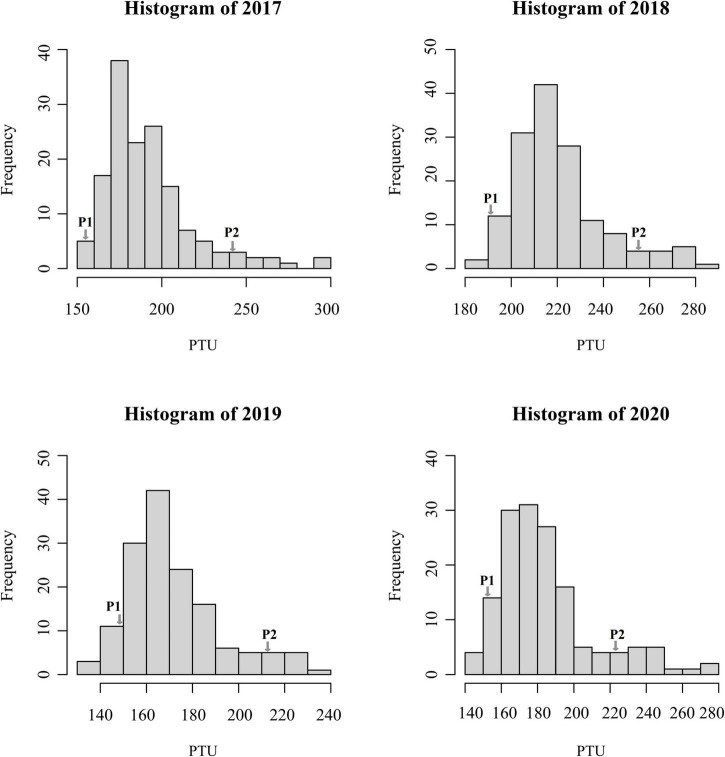
Frequency distributions of photothermal unit values in the F_1_ population at Langfang, Hebei Province from the year 2017 to 2020.

**FIGURE 2:**
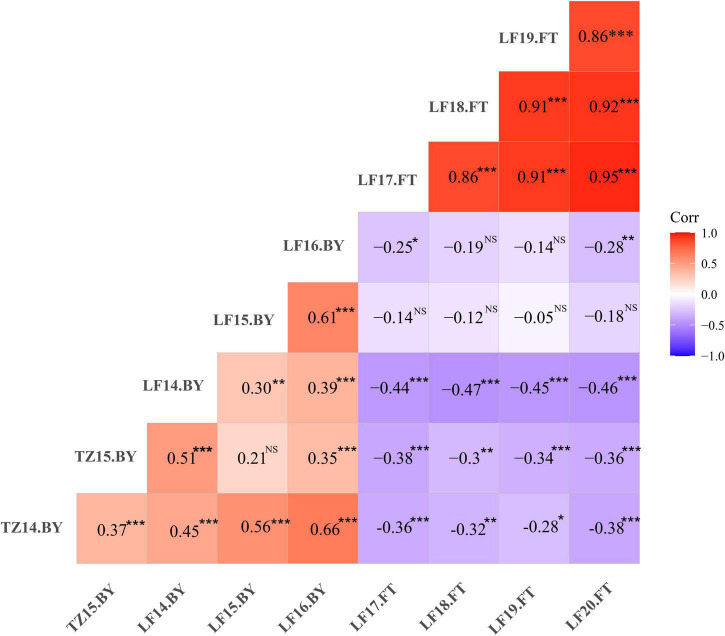
Phenotypic correlations of flowering time (FT) and biomass yield (BY) among different environments in the F_1_ population. Red and blue colors represent positive and negative correlations (*r*) between traits, respectively. TZ; Tongzhou; LF, Langfang, e.g., TZ14.BY indicates biomass yield data collected at Tongzhou in 2014. *P < 0.05, **P < 0.01, ***P < 0.001, NS, non-significant. The results of yield were published in our previous study Zhang et al. (2019).

Phenotypic data of the F_1_ population and parents were collected in Langfang, Hebei Province for four consecutive years from 2017 to 2020. Our previous work showed that P1 and P2 differed in yield and leaf size (He et al., 2019; Zhang et al., 2019). In addition, FT appeared to differ considerably (Table 1), suggesting that these two parents represent substantial variations among alfalfa varieties. Phenotypic variations in FT among the F_1_ population ranged from 11.28 to 16.00% (Table 1), and transgressive segregation indicated the FT trait was quantitatively inherited. The frequency distribution of FT revealed a nearly normal distribution for FT trait in all 4 years (Figure 1). In our previous study, the same mapping population was used to identify QTLs associated with biomass yield (Zhang et al., 2019). A weak negative correlation was observed between FT and yield in the mapping population with correlation coefficients ranging from −0.05 to −0.47 (Figure 2), indicating that the genotypes with earlier flowering time (shorter vegetative growth time) tended to have lower yields. The result was similar to that of a previous study on alfalfa (Adhikari et al., 2019). The *H*^2^ of FT ranged from 0.68 to 0.78 (Table 1). The relatively high heritability suggested that genotypic variance accounted for a high proportion of total phenotype variance for FT in this mapping population.

**TABLE 1:** Statistical analysis of LS means of photothermal units values in parents and F1 population across 4 years.

Year	Parents		F_1_ population				H^2^
	P1	P2	Range	Mean	CV (%)	Se	
2017	15.44	242.94***	150.77∼293.40	192.01	14.47	2.13	0.74
2018	191.65	256.78**	185.23∼288.11	221.06	11.28	1.61	0.71
2019	49.56	213.72**	135.46∼233.17	171.52	15.15	1.64	0.68
2020	153.06	223.16*	143.15∼271.69	184.61	16.00	2.18	0.78

CV, Coefficient of variance; se, Standard error; H2, broad-sense heritability. Asterisks indicate significant differences in FT between parents (t-test, *P < 0.05; **P < 0.01; ***P < 0.001).

### Genetic Linkage Map Construction

**FIGURE 3:**
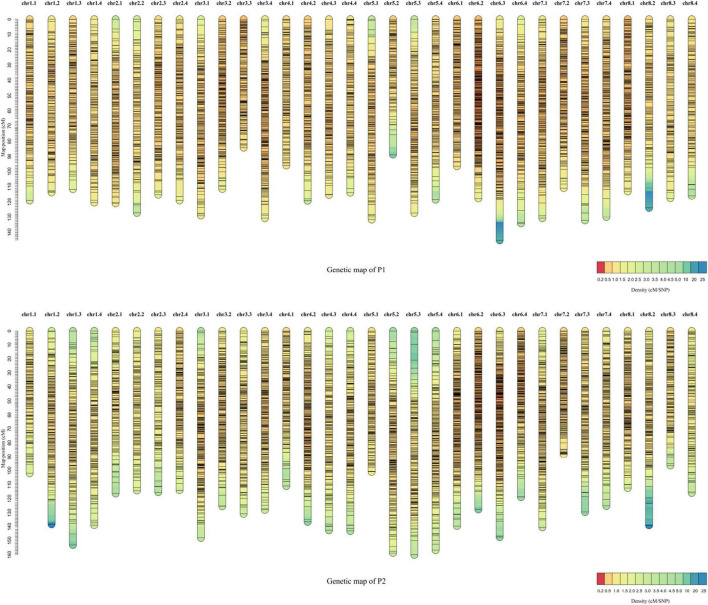
Genetic linkage maps for the F_1_ population. Distribution of 6,844 and 6,929 single-dose allele (SDA) markers in the parent (P1) and parent (P2) genetic maps, respectively. *X*-axis is the chromosome number and *Y*-axis describes the CentiMorgan distance. Detailed information on the maps is listed in Supplementary Table 1.

Each F_1_ progeny was subjected to RAD-seq to detect genome-wide SNPs. A total of 161,169 raw SNPs were detected by aligning the sequences to the XinJiangDaYe reference genome (Chen H. et al., 2020). SNPs were filtered with mean read depth < 5, missing rate > 20%, heterozygosity ≥ 2:1. After filtering, 7,252 and 7,404 high-quality SDA markers were obtained for P1 and P2 parents, respectively; which were then used to construct high-density linkage maps. The total genetic lengths were 3780.49 cM for P1 and 4113.45 cM for P2 maps (Supplementary Table 1). The P1 linkage maps consisted of 6,844 SDA markers with an average genetic distance of 0.58 cM and a map length from 84.30 (chr3.3) to 145.22 cM (chr6.3) (Supplementary Tables 1–3 and Figure 3). In P1, the lowest marker density was 0.85 on chr2.2 and the highest was 0.31 on chr6.2 (Supplementary Table 1 and Figure 3). The P2 linkage maps contained 6,929 SDA markers with an average genetic distance of 0.59 cM, and a map length from 88.19 (chr7.2) to 160.14 cM (chr5.3) (Supplementary Table 1 and Figure 3). Among the 32 linkage groups of P2, the lowest marker density was 0.89 on chr2.3 and the highest was 0.36 on chr6.2 (Supplementary Table 1 and Figure 3).

### Quantitative Trait Loci Mapping for Flowering Time

Using the ICIM-ADD mapping method, the LS means of each year and BLUP values across 4 years were used for QTL mapping. A total of 16 QTLs for FT were detected for P1 (Table 2 and Supplementary Figure 1). The percentage of phenotypic variance explained (PVE) by each QTL was from 4.13 to 16.04%, with the highest PVE observed on *qFT7.4-3*. Twenty-two QTLs for FT were detected for P2 parent with PVE values ranging from 1.40 to 13.78% and the highest PVE was observed on *qFT2.3-2* (Table 2 and Supplementary Figure 2).

**TABLE 2:** QTLs detected for least-square means (LS means) and the best linear unbiased prediction (BLUP) values of flowering time (FT) using ICIM-ADD in the F1 population.

Parent	QTL	Year	Chr.	Location (cM)	Left marker	Right marker	LOD	PVE (%)	Add
	*qFT1.2-1*	2020	1.2	81.5∼82.5	chr1.2__77498697	chr1.2__55012028	4.06	6.33	15.34
	*qFT1.4*	2017	1.4	33.5∼34.5	chr1.4__9568849	chr1.4__8751633	3.59	4.56	−12.30
	*qFT2.3-1*	BLUP	2.3	78.5∼79.5	chr2.3__58327605	chr2.3__51410345	4.84	6.10	−9.99
	*qFT3.3-1*	BLUP	3.3	50.5∼51.5	chr3.3__62421973	chr3.3__69012912	3.83	4.13	−8.38
	***qFT4.1-1***	2017, BLUP	4.1	63.5∼64.5	chr4.1__64642892	chr4.1__77321616	4.06, 5.82	6.68, 12.43	−15.04, −14.43
	*qFT4.1-2*	2017	4.1	92.5∼93.5	chr4.1__75331077	chr4.1__75711577	4.43	5.43	−13.60
	*qFT4.2*	BLUP	4.2	9.5∼10.5	chr4.2__34610643	chr4.2__11026558	3.06	4.46	8.57
P1	***qFT5.2-1***	2017, 2020, BLUP	5.2	4.5∼5.5	chr5.2__45638433	chr5.2__44056158	6.71, 5.48, 5.32	6.99, 5.83, 6.13	−15.25, −14.74, −10.04
	*qFT5.2-2*	2018	5.2	34.5∼35.5	chr5.2__57773538	chr5.2__56102093	4.83	6.23	11.26
	*qFT6.1*	BLUP	6.1	76.5∼77.5	chr6.1__44126752	chr6.1__76961406	5.73	8.14	11.71
	***qFT6.3-1***	2017, 2019, 2020	6.3	95.5∼96.5	chr6.3__52410664	chr6.3__61869379	12.03, 8.97, 4.91	14.37, 15.39, 6.32	21.86, 17.05, 15.31
	*qFT7.3-1*	2017	7.3	11.5∼13.5	chr7.3__8960937	chr7.3__12357404	3.28	4.28	11.91
	*qFT7.3-2*	2019	7.3	64.5∼65.5	chr7.3__63489699	chr7.3__50361610	4.51	7.2	11.65
	***qFT7.4-1***	2017, 2020, BLUP	7.4	62.5∼63.5	chr7.4__62411379	chr7.4__61234796	4.91, 3.82, 5.85	7.63, 6.52, 10.78	16.06, 15.64, 13.41
	*qFT7.4-2*	2018, 2020	7.4	17.5∼18.5	chr7.4__35788679	chr7.4__40755488	5.01, 8.18	8.02, 11.09	−12.91, −20.55
	*qFT7.4-3*	2018	7.4	93.5∼94.5	chr7.4__87919782	chr7.4__85044135	7.27	16.04	−18.10

	*qFT1.1-1*	2017	1.1	68.5∼69.5	chr1.1__75472691	chr1.1__70841814	3.22	3.54	−10.00
	*qFT1.1-2*	2019	1.1	50.5∼51.5	chr1.1__45209340	chr1.1__56558159	4.40	2.25	6.80
	*qFT1.1-3*	BLUP	1.1	9.5∼10.5	chr1.1__22559325	chr1.1__16294404	4.22	6.54	11.18
	*qFT1.2-2*	2017, 2019	1.2	91.5∼92.5	chr1.2__71070950	chr1.2__55221961	5.18, 18.81	5.75, 9.08	−13.21, −13.37
	*qFT1.2-3*	2020	1.2	104.5∼105.5	chr1.2__71071980	chr1.2__81663663	3.98	9.79	−17.22
	*qFT1.3*	2019	1.3	101.5∼102.5	chr1.3__64152948	chr1.3__65763782	11.64	9.47	−13.28
	*qFT2.3-2*	2017, 2019, 2020, BLUP	2.3	78.5∼79.5	chr2.3__59083503	chr2.3__59862317	8.66, 16.26, 3.89, 6.46	13.56, 9.44, 9.94, 13.78	−20.02, −13.42, −17.72, −16.07
P2	*qFT2.3-3*	2018	2.3	64.5∼65.5	chr2.3__46634301	chr2.3__56553524	4.12	7.45	−13.13
	*qFT3.2-1*	2019	3.2	23.5∼24.5	chr3.2__5624964	chr3.2__29030021	4.36	4.80	9.46
	*qFT3.2-2*	2019	3.2	104.5∼105.5	chr3.2__90944356	chr3.2__78402762	8.63	3.8	−8.79
	*qFT3.2-3*	BLUP	3.2	53.5∼54.5	chr3.2__41429169	chr3.2__37443429	3.64	8.61	−12.56
	*qFT3.3-2*	2019	3.3	15.5∼16.5	chr3.3__13703202	chr3.3__12599738	4.46	2.48	−6.80
	*qFT4.1-3*	2019	4.1	64.5∼65.5	chr4.1__63974266	chr4.1__80043069	11.04	5.04	−9.83
	*qFT4.3*	2017	4.3	97.5∼98.5	chr4.3__49979397	chr4.3__58477555	3.66	4.66	−11.46
	*qFT5.1*	2019	5.1	9.5∼10.5	chr5.1__20167931	chr5.1__18706007	5.87	2.97	7.53
	*qFT5.2-3*	2017	5.2	142.5∼144.5	chr5.2__82957406	chr5.2__82957429	6.96	7.71	14.73
	*qFT5.2-4*	2019	5.2	141.5∼142.5	chr5.2__80311479	chr5.2__82957410	6.10	2.66	6.99
	*qFT6.3-2*	2019	6.3	28.5∼29.5	chr6.3__5222282	chr6.3__5981497	12.64	5.72	−10.45
	*qFT6.3-3*	2019	6.3	33.5∼34.5	chr6.3__5307300	chr6.3__6965213	3.81	2.45	6.71
	*qFT7.3-3*	2019	7.3	59.5∼60.5	chr7.3__38899574	chr7.3__63369976	5.59	6.83	−11.60
	*qFT7.4-4*	2017, 2018, 2019, 2020, BLUP	7.4	39.5∼40.5	chr7.4__13015502	chr7.4__51199977	5.05, 5.32, 11.26, 3.45, 4.15	7.42, 10.99, 6.97, 9.37, 9.61	14.46, 15.74, 11.35, 16.80, 13.10
	*qFT8.3*	2017, 2019, 2020, BLUP	8.3	87.5∼88.5	chr8.3__75627434	chr8.3__81295867	6.89, 3.68, 3.48, 3.62	7.83, 1.40, 6.41, 5.76	−14.93, −5.10, −13.94, −10.17

*Sixteen and Twenty-one QTLs for FT were mapped on P1 and P2 linkage maps, respectively. Consistent QTLs were underlined; Novel QTLs were bold. Left marker, the marker on the left of the LOD peak; right marker, the marker on the right of the LOD peak; LG, linkage group; interval (cM), 1-LOD support interval; LOD, the logarithm of the odds; PVE, the percentage of the phenotypic variation explained by QTL; Add, the additive effects of the QTL.*

Five QTLs (*qFT4.1-1*, *qFT5.2-1*, *qFT6.3-1*, *qFT7.4-1*, and *qFT7.4-2*) in P1 parent and four QTLs (*qFT1.2-2*, *qFT2.3-2*, *qFT7.4-4*, and *qFT8.3*) in P2 parent were independently co-detected in multi-environments (Table 2). In P1, *qFT6.3-1* was identified in three grown environments (2017, 2019, and 2020) on chr6.3 at a position of 95.5–96.5 cM with an average PVE value of 12.03% (Table 2). Three QTLs (*qFT7.4-1*, *qFT7.4-2*, and *qFT7.4-4*) were identified on chr7.4 across multiple environments. Of them, *qFT7.4-4* on P2 was detected in all 4 years and BLUP values with an average LOD value of 5.85 and a PVE value of 8.87 (Table 2).

### Novel Environment-Stable Quantitative Trait Locis Controlling Flowering Time

Four intervals were novel signals as they were detected in more than one environment and did not overlap with previously known FT-related QTLs. All of them were identified in the P1 parent, including *qFT4.1-1*, *qFT5.2-1*, *qFT6.3-1*, and *qFT7.4-1* (Table 2). The average PVE value of these four loci were 9.56, 6.32, 12.03, and 8.31%. Among them, *qFT6.3-1* was identified in three environments (2017, 2019, and 2020) with the highest average PVE value of 12.03% and an average LOD value of 8.64.

### Consistent Quantitative Trait Locis Controlling Flowering Time

Four novel QTLs for FT such as *qFT4.1-1, qFT5.2-1, qFT6.3-1*, and *qFT7.4-1* were detected in this study, enriching our current knowledge of genetic control of FT in alfalfa. We also detected seven QTLs that overlapped with known QTLs. These QTLs, two from P1 and five from P2, may be considered as consistent QTLs (Table 2). Specifically, *qFT1.1-2* located at 45.2–68.9 Mb on chr1.1 was in the same region as *qflower-5* and *Tof-d1* (Adhikari et al., 2019; Zhang et al., 2020), while *qFT1.2-1* and *qFT1.2-2* fell into a known region of 55.0–77.5 Mb with *qflower-20*, *Tof-d6*, *Tof-d10*, and *Tof-d2* on chr1.2 (Adhikari et al., 2019; Zhang et al., 2020). On chr2.3, *qFT2.3-2* was overlapped with *qflower-6* (Zhang et al., 2020). The physical position of *qFT7.4-4* was relatively large (13.0 ∼ 51.2 Mb), and overlapped with *qFT7.4-2*, *Tof-d5*, *Tof-d4*, and *Tof-n6* (Adhikari et al., 2019). *qFT8.3* was located at 66.9–82.1 Mb on chr8.3, overlapped with *qflower-28* (Zhang et al., 2020). The results demonstrated that these QTLs could be robust signals for genetic control of FT in alfalfa. Other QTLs were detected in only one environment with a lower LOD value, suggesting that these QTLs may be environment-specific.

### Integration of Differentially Expressed Genes With Quantitative Trait Locis

**FIGURE 4:**
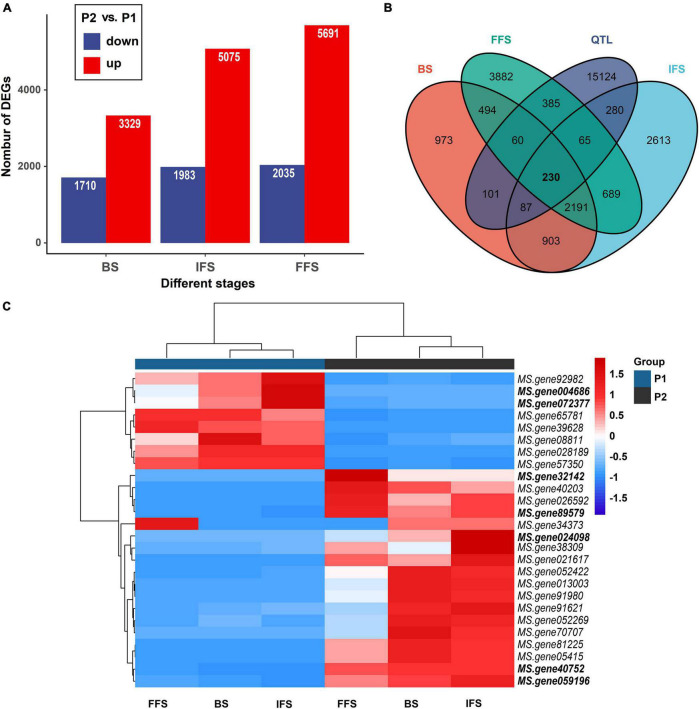
Differentially expressed genes (DEGs) within QTL intervals. **(A)** Number of downregulated genes (blue) and upregulated genes (red) between two parents at three flowering stages. BS, budding stage, IFS, initial flowering stage and FFS, full flowering stage. **(B)** The number of DEGs within QTL intervals. **(C)** Heatmap clustering the DEGs associated with flowering time by their expression abundance. DEGs within the consistent QTL intervals or new environment-stable QTLs were bold.

**FIGURE 5:**
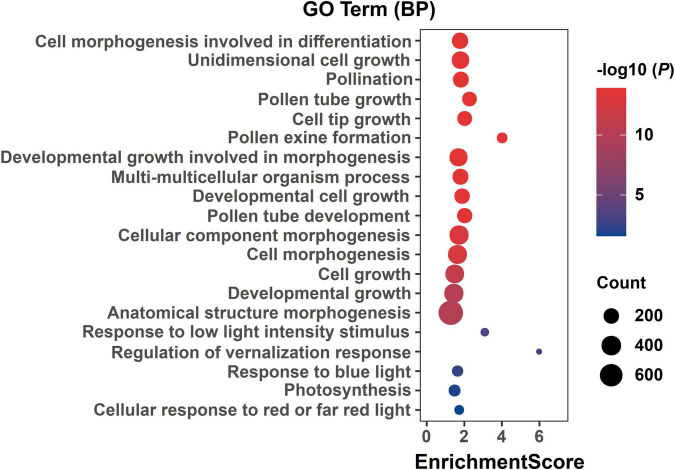
GO enrichment of DEGs in three alfalfa flowering stages. The ordinate represents different GO terms (biological progress) and the abscissa represents enrichment Score. Circle size represents the gene number while circle color represents the value of −log10 (*P*). The *P*-value was corrected by Benjamini and Hochberg (BH) method.

A larger number of significant DEGs were found at three flowering stages between two parents (P2 vs. P1). At BS, between P1 and P2, 3,329 and 1,710 DEGs were up- and downregulated, respectively (Figure 4A). At IFS, between P1 and P2, 5,075 and 1,983 DEGs were up- and down-regulated, respectively (Figure 4A). Between P1 and P2 at FFS, a total of 5,691 and 2,305 DEGs were up- and down-regulated, respectively (Figure 4A). These DEGs in three alfalfa flowering stages were then categorized into the biological process (BP), cellular component (CC), and molecular function (MF) categories using GO enrichment analysis (Supplementary Table 5). Specifically, in the BP terms, DEGs were mainly enriched in “cell morphogenesis involved in differentiation,” “pollen tube growth,” “developmental growth involved in morphogenesis,” “anther morphogenesis,” and “stamen morphogenesis.” Also, these DEGs had a role in “response to low light intensity stimulus,” “regulation of vernalization response,” and “response to blue light” among others (Supplementary Table 5 and Figure 5). The results suggested that these DEGs may play a role in the regulation of floral organ development and flowering time.

**FIGURE 6:**
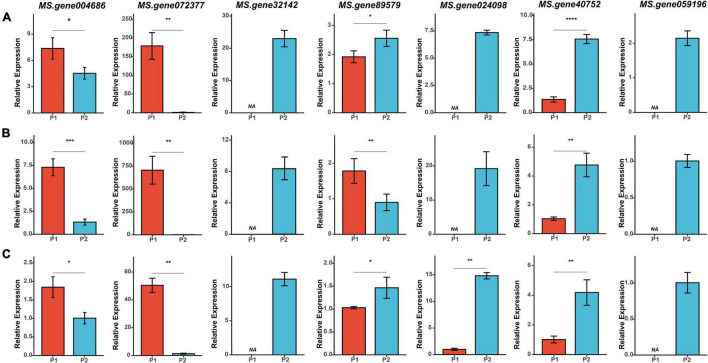
qRT-PCR analysis of the seven candidate genes at three flowering stages in two parents. **(A–C)** Relative expression of the seven candidate genes at BS (budding stage), IFS (initial flowering stage), and FFS (full flowering stage), respectively. Asterisk symbols indicate significant differences between two parents (*t*-test, *p < 0.05; **p < 0.01; ***p < 0.001, ****P < 0.0001).

A comparison of the results from QTL mapping and RNA-seq analysis was employed to reveal candidate genes regulating the FT of alfalfa. A total of 230 common DEGs at three flowering stages were found in the QTL intervals (Supplementary Table 6 and Figure 4B). Of which, 26 DEGs were annotated as candidate genes associated with flowering time in plants (Figure 4C). Interestingly, seven candidates were co-localized within consistent QTLs or novel environment-stable QTLs. Among them, six DEGs resided in three consistent QTL regions of *qFT1.1-2* (*MS.gene89579* and *MS.gene004686*), *qFT1.2-1* and *qFT1.2-2* (*MS.gene32142* and *MS.gene40752*), *qFT7.4-2* and *qFT7.4-4* (*MS.gene072377* and *MS.gene024098*). The last one, *MS.gene059196*, was found in a novel environment-stable QTL region of *qFT4.1-1*. The relative gene expression levels of the seven candidates were validated by qRT-PCR. The results showed that RNA-seq and qRT-PCR results were highly consistent and the seven candidate genes were differently expressed in two parents at three flowering stages (Figure 6).

## Discussion

Large phenotypic variations of FT were observed in the F_1_ mapping population. The range of FT was comparable to a previous study identified in alfalfa population (Zhang et al., 2020). Alfalfa genotypes with late flowering showed an enhanced biomass yield (Adhikari et al., 2019). Our results also demonstrated that biomass yield was negatively correlated with FT in the F_1_ mapping population. Large phenotypic variations and the high heritability of FT provide an important basis for analyzing QTL mapping in alfalfa.

Although FT-related QTLs have been reported in previous studies (Adhikari et al., 2019; Zhang et al., 2020), there were some limitations due to the mapping population and the lack of a reference genome. For example, FT-related QTLs were identified in a pseudo-testcross F_1_ population derived from cultivars 3010 and CW 1010, but the two parents did not show significant differences in FT traits. Moreover, the flowering date was recorded as the day of the year (e.g., January 1st = 1) and was only recorded every 3 days (Adhikari et al., 2019). In addition, the genetic linkage maps were previously constructed by SNP markers generated through genotype−by−sequencing, not RAD-seq (Adhikari et al., 2019; Zhang et al., 2020). This could result in some differences in marker density and quality for creating genetic linkage maps. In this study, the two parental materials greatly varied for FT, and genome-wide SDA markers were detected with XinJiangDaYe reference genome. Therefore, our results on the constructed genetic maps and identified QTLs would add additional values to a better understanding of the genetic control of FT in alfalfa.

### High-Density Genetic Linkage Maps

The creation of a high-density and quality linkage map is an important prerequisite for QTL mapping and map-based cloning of genes. Alfalfa is autotetraploid and highly heterozygous, which has impeded the construction of a high-density linkage map (Li et al., 2014). An early study reported that a tetraploid alfalfa linkage map had seven linkage groups with 443 cM because of low marker density (Brouwer and Osborn, 1999). Powerful new sequencing techniques can generate a large number of SNP markers for linkage map construction (Andolfatto et al., 2011; Yang et al., 2012). Li et al. (2014) first used SDA markers to construct high-density linkage maps for two alfalfa parents to identify QTLs associated with important agronomic traits. Their genetic maps with 64 linkage groups contained 3,591 SDA markers, with an average density of one marker per 1.5 and 1.0 cM for the maternal and paternal parents, respectively (Li et al., 2014). However, the deficiency of genomic information has been the major limitation for genetic map construction. The first available alfalfa genome sequence makes it possible to construct high-density genetic maps (Chen H. et al., 2020). In this study, the maps contained 13,773 SDA markers on 64 linkage groups across both parents, with an average marker distance of 0.58 and 0.59 cM for the P1 and P2 maps, respectively. These maps will be useful for future QTL mapping studies and the application of marker-assisted selection (MAS).

Population size and quality of linkage map greatly influence the power of QTL detection. Adhikari et al. (2019) performed QTL mapping for the flowering time by using a population of 181 F1 progenies in four environments. The heritability of flowering phenotypes in three of these environments (0.69, 0.74, and 0.75) was similar to our study, while only three QTLs individually co-localized in two environments. In a population of 392 F1 progenies, Zhang et al. (2020) identified three environment-stable QTLs, with one found in three environments and two found in two environments. In this study, we identified eight environment-stable QTLs, suggesting that our population size was big enough for QTL identification. Moreover, the high-density linkage map constructed in this study had an increased power of QTL detection compared with maps developed in previous studies (Adhikari et al., 2019; Zhang et al., 2020). Nonetheless, in our study, a major limitation remains due to the only use of SDA markers. Many polymorphic SNP markers not classified as SDA were not used for the genetic map construction, resulting in the loss of genetic information and the reduced power of QTL detection (Li et al., 2014). As a result, despite the high heritability of flowering time, only one environment-stable QTL was discovered across all four environments in our study.

### Analysis of Candidate Genes for Flowering Time

Previous studies into FT mainly focused on QTL mapping in alfalfa (in genomics level) (Adhikari et al., 2019; Zhang et al., 2020). Although integration of QTL mapping and RNA-seq has been applied to identify candidate genes in rice, maize (*Zea mays* L.), wheat (*Triticum aestivum* L.), and other crops (Kuang et al., 2020; Lei et al., 2020; Han et al., 2022), but such approaches have not been used for discovering alfalfa FT genes. In this study, flower samples at three flowering stages from two parents of the F_1_ mapping population were characterized using the RNA-seq technology, and the identified DEGs were combined with QTLs to narrow the candidates. Finally, based on DEG annotations within QTL intervals, seven potential genes associated with flowering time were detected.

Two candidates, *MS.gene89579* and *MS.gene004686*, located within qFT1.1-2. *MS.gene89579*, was annotated as a casein kinase I-like protein, which was previously reported to function in regulating flowering time through gibberellin signaling (Hori et al., 2013; Kang et al., 2020). Another one, *MS.gene004686*, was annotated as a basic helix-loop-helix (BHLH) transcription factor. Its homologous gene in Arabidopsis has been reported to control flowering time in response to blue light (Liu et al., 2013). In the interval of *qFT1.2-1* and *qFT1.2-2*, two candidates (*MS.gene32142* and *MS.gene40752*) associated with flowering time were annotated. *MS.gene32142* homolog was ethylene receptor ETR2, which can delay flowering and cause starch accumulation in stems (Wuriyanghan et al., 2009). *MS.gene40752* homolog was glycogen synthase kinase 3, which played an important role in preventing precocious flowering through phosphorylating CO (Chen Y. et al., 2020). In the physical interval of two consistent QTLs (*qFT7.4-2* and *qFT7.4-4*), *MS.gene072377* and *MS.gene024098* were identified as candidate genes. *MS.gene072377*, annotated as leucine-rich repeat protein FLOR 1, promoted flowering transition in long days (Torti et al., 2012). *MS.gene024098* was a member of UDP-glucosyltransferase family, and its homologous gene can regulate flowering time via *flowering locus C* (Wang et al., 2012). In the physical interval of qFT4.1-1, *MS.gene059196* was annotated as a pleiotropic regulatory locus. Its mutants (prl1) caused an early flowering phenotype in Arabidopsis (Weihmann et al., 2012).

In plants, the *FLOWERING LOCUS T* (FT) family of genes is known to be associated with photoperiod sensing, which is involved in the initiation of flowering. By functional annotation, two homologues of *FT*, *MS.gene51911* and *MS.gene87044*, were identified in a consistent QTL interval (*qFT7.4-2* and *qFT7.4-4*). They were annotated as *FTc* and *FTb*, respectively. Interestingly, *MsFTb1* could partially complement the phenotype of late flowering mutants (*ft-10*), while *MsFTc* had no effect on the flowering time in alfalfa (Lorenzo et al., 2020). The above-mentioned genes in the QTL region were not among the DEGs detected by RNA-Seq analysis, potentially due to the specific expression of FT genes in leaves (Huang et al., 2005).

## Conclusion

Plant flowering is regulated by genetic and environmental signals. The work presented here revealed the genetic mechanisms controlling flowering time in the alfalfa F_1_ population through QTL mapping. High-density genetic maps were created and 38 QTLs for FT were detected, including seven consistent QTLs and four novel environment-stable QTLs. Seven candidate genes involved in flowering regulation were annotated by QTL mapping and DEGs analysis. The functions of these candidate genes could be further verified in alfalfa. The loci generated by the current QTL mapping study are valuable resources for enhancing breeding programs aimed at improving flowering time and yield in alfalfa.

## Data Availability Statement

The datasets presented in this study can be found in online repositories. The names of the repository/repositories and accession number(s) can be found below: https://www.ncbi.nlm.nih.gov/bioproject/PRJNA817856.

## Author Contributions

QY, JK, and ZW conceived, designed the experiments, and developed the mapping population. XJ, FH, XY, and CY collected phenotypic data. XJ, FZ, TY, and TG performed data analysis. XJ and ZW wrote the manuscript. YJ and JK revised and finalized the manuscript. All authors read and approved the final manuscript.

## Conflict of Interest

The authors declare that the research was conducted in the absence of any commercial or financial relationships that could be construed as a potential conflict of interest.

## Publisher’s Note

All claims expressed in this article are solely those of the authors and do not necessarily represent those of their affiliated organizations, or those of the publisher, the editors and the reviewers. Any product that may be evaluated in this article, or claim that may be made by its manufacturer, is not guaranteed or endorsed by the publisher.

## Footnotes

1
http://tianqi.2345.com/wea_history/60198.htm

2
https://richurimo.51240.com/beijing__richurimo/

3
www.kyazma.nl

4
www.isbreeding.net

5
https://www.ncbi.nlm.nih.gov/bioproject/PRJNA817856

6
https://www.bioinformatics.babraham.ac.uk/projects/fastqc/

7
https://figshare.com/projects/whole_genome_sequencing_and_assembly_of_Medicago_sativa/66380

8
https://www.ncbi.nlm.nih.gov/

9
https://ensembl.gramene.org/

10
http://eggnog-mapper.embl.de/

## Supplementary Material

Supplementary Figure 1 Distribution of QTLs for flowering time (FT) using LS means of each environment and BLUP values in P1 parent.

Supplementary Figure 2 Distribution of QTLs for flowering time (FT) detected using LS means of each environment and BLUP values in P2 parent.

Supplementary Table 5 GO enrichment analysis of the DEGs.
