# Supplementary figures and images for: RAD-Seq-Based High-Density Linkage Maps Construction and Quantitative Trait Loci Mapping of Flowering Time Trait in Alfalfa (Medicago sativa L.)
Source: Front Plant Sci. 2022 May 26;13:899681. doi: 10.3389/fpls.2022.899681 (PMC9199863; doi:10.3389/fpls.2022.899681)

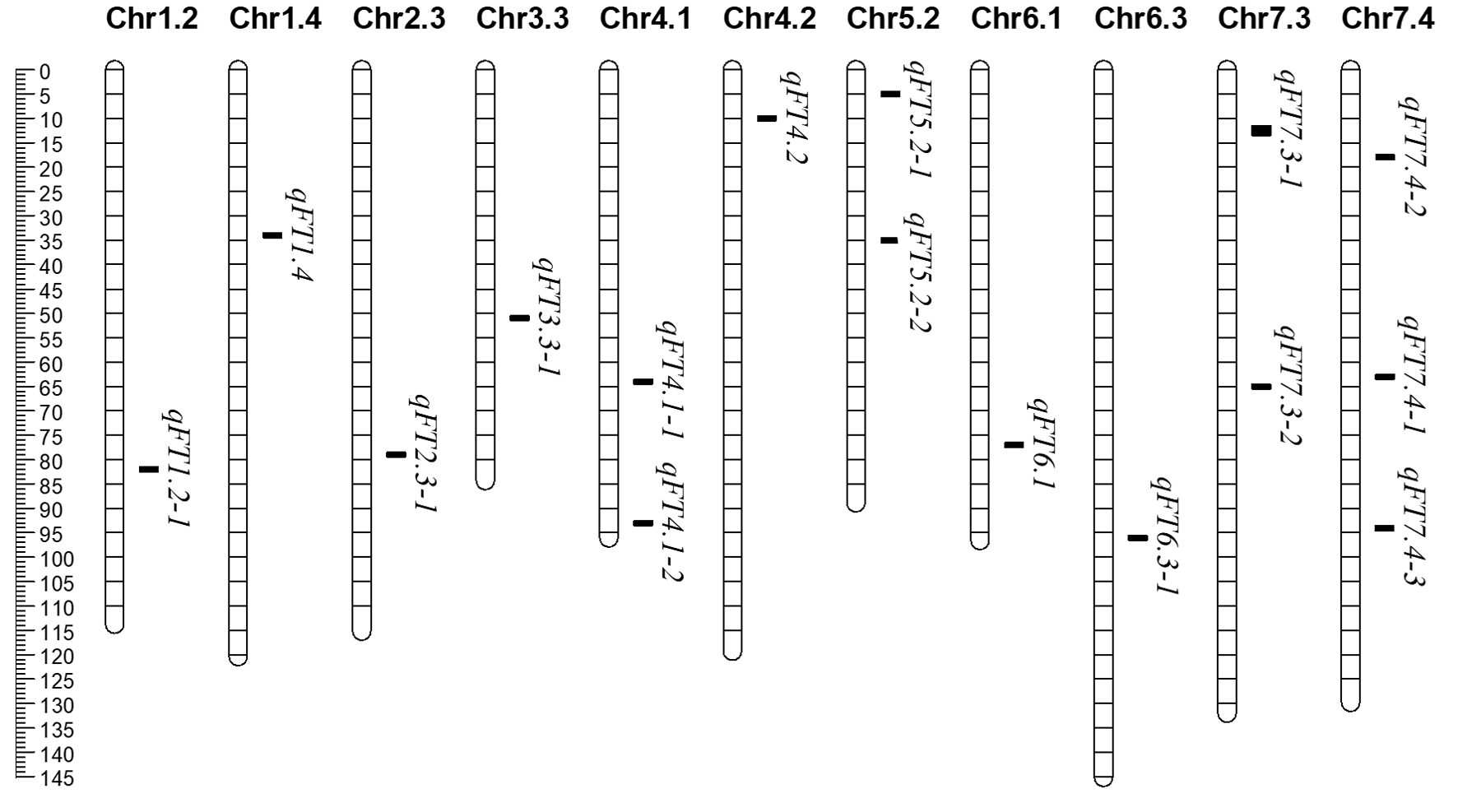

Supplement: Supplementary Figure 1 — Distribution of QTLs for flowering time (FT) using LS means of each environment and BLUP values in P1 parent. [file Image_1.TIF]

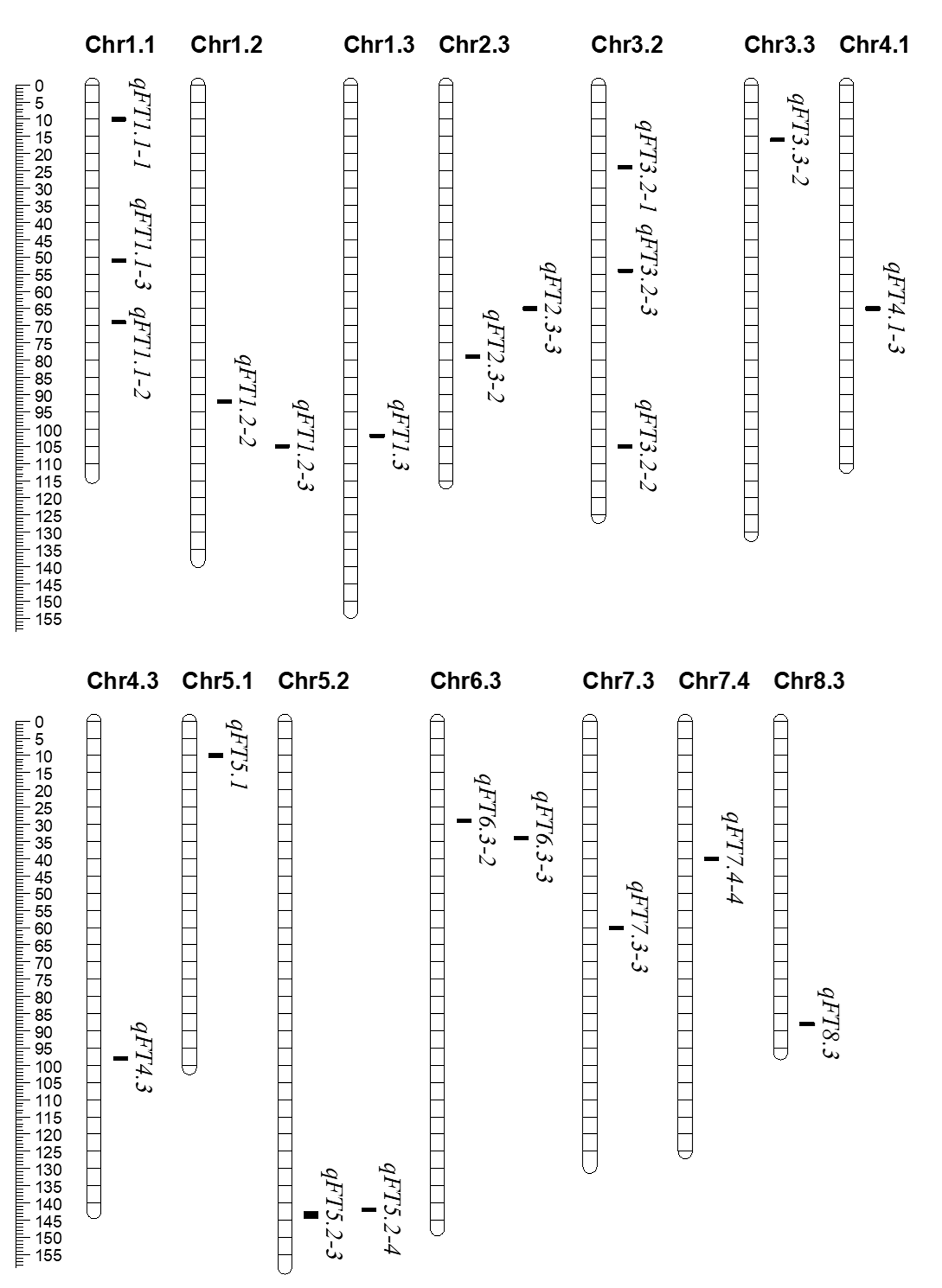

Supplement: Supplementary Figure 2 — Distribution of QTLs for flowering time (FT) detected using LS means of each environment and BLUP values in P2 parent. [file Image_2.TIF]
